# 

**Published:** 1894-04

**Authors:** 


					﻿Vol Xiv.
APRIL, 1894.
NO. 4/
THE
JIOMtEOPATHlC PHYSICIAN,
A Monthly Journal of Homoeopathic Materia Medica
and Clinical Medicine.
•* He it a freeman whom truth makes free, and all are tlaves betide."
"Seek the Truth; come whence it may, cost what it will."
EDITED AND PUBLISHED BY
WALTER M. JAMES, M. D.
PHILADELPHIA :
No. 1125 SPRUCE STREET.
zo w d o n :
ALFRED HEATH & CO., Bole Agents fob Gbbat Britain,
114 Ebury Street, Eaton Square.
S&* Yearly Subscription, $2.SO, invariably in advance. Single numbers, 2S cts.
Entered at the Philadelphia Peet-Offiee ae Beoond Claae Mall Matter.
				

